# Effects of leptin on in vitro maturation, fertilization and embryonic cleavage after ICSI and early developmental expression of leptin (Ob) and leptin receptor (ObR) proteins in the horse

**DOI:** 10.1186/1477-7827-7-113

**Published:** 2009-10-16

**Authors:** Anna Lange Consiglio, Maria Elena Dell'Aquila, Nadia Fiandanese, Barbara Ambruosi, Yoon S Cho, Giampaolo Bosi, Silvana Arrighi, Giovanni M Lacalandra, Fausto Cremonesi

**Affiliations:** 1 Università degli Studi di Milano, Reproduction Unit, Large Animal Hospital, Faculty of Veterinary Medicine, Via dell'Università 6,-26900 Lodi, Italy; 2 Department of Animal Production, Faculty of Biotechnological Sciences, S Prov Casamassima, km 3 - 70010 Valenzano (Bari), Italy; 3 Department of Veterinary Science and Technologies for Food Safety, Laboratory of Anatomy, - via Trentacoste, 2 - 20134 Milano, Italy; 4 Assisted Procreation Unit, Clinica Santa Maria, Bari, Italy

## Abstract

**Background:**

The identification of the adipocyte-derived obesity gene product, leptin (Ob), and subsequently its association with reproduction in rodents and humans led to speculations that leptin may be involved in the regulation of oocyte and preimplantation embryo development. In mice and pigs, in vitro leptin addition significantly increased meiotic resumption and promoted preimplantation embryo development in a dose-dependent manner. This study was conducted to determine whether leptin supplementation during in vitro maturation (IVM) to horse oocytes could have effects on their developmental capacity after fertilization by IntraCytoplasmic Sperm Injection (ICSI).

**Methods:**

Compact and expanded-cumulus horse oocytes were matured in medium containing different concentrations (1, 10, 100, 1000 ng/ml) of recombinant human leptin and the effects on maturation, fertilization and embryo cleavage were evaluated. Furthermore, early developmental expression of Ob and leptin receptor (Ob-R) was investigated by immunocytochemical staining.

**Results:**

In expanded-cumulus oocytes, the addition of leptin in IVM medium improved maturation (74% vs 44%, for 100 ng/ml leptin-treated and control groups, respectively; P < 0.05) and fertilization after ICSI (56% vs 23% for 10 ng/ml leptin-treated and control groups, respectively; P < 0.05). However, the developmental rate and quality of 8-cell stage embryos derived from leptin-treated oocytes (100 ng/ml) was significantly reduced, in contrast to previous data in other species where leptin increased embryo cleavage. Ob and Ob-R proteins were detected up to the 8-cell stage with cortical and cytoplasmic granule-like distribution pattern in each blastomere.

**Conclusion:**

Leptin plays a cumulus cell-mediated role in the regulation of oocyte maturation in the mare. Species-specific differences may exist in oocyte sensitivity to leptin.

## Background

Leptin, the product of the obesity (Ob) gene [1], predominantly synthesized by adipocytes, has been shown to be involved in the regulation of the reproductive function [2] and recent studies have been performed, by exploiting the potential role of this hormone in animal models, such as mouse, swine and bovine, to evaluate the possibility of improving *in vitro *oocyte maturation and embryo culture procedures. In the mouse, Kawamura et al. [3,4] demonstrated that leptin supplementation in the culture medium (10, 100 and 1000 ng/ml) promoted embryo development and increased the cell numbers of cultured blastocysts and the effect was preferentially observed in the trophoectoderm. These findings raised the possibility that leptin might regulate mouse preimplantation embryo development through a paracrine pathway. In pigs, leptin addition in oocyte maturation medium (10 and 100 ng/ml) significantly increased the proportion of oocytes reaching the metaphase II (MII) stage, elevated ooplasmic cyclin B1 protein content and enhanced embryo developmental potential, thus suggesting that leptin might play a role in both nuclear and cytoplasmic maturation [5]. During porcine oocyte maturation, leptin increased phosphorylated mitogen-activated protein kinase (MAPK) content by 2.8-fold, and leptin-stimulated oocyte maturation was blocked when leptin-induced MAPK phosphorylation was suppressed by a specific MAPK activation inhibitor, U0126, demonstrating that leptin enhanced nuclear maturation via activation of the MAPK pathway [5]. Kun et al. [6] confirmed that 10 and 100 ng/ml of leptin in maturation medium enhanced porcine embryo development. These authors showed that there was no effect of the timing of leptin supplementation, in maturation medium, on meiotic maturation of porcine oocytes. In bovine, Paula-Lopes et al. [7] showed that leptin supplementation (1 and 10 ng/ml) exerted positive effects during oocyte maturation, by influencing blastocyst development, apoptotic index in cumulus cells and transcript levels of developmentally important genes. Moreover, they demonstrated a role for cumulus cells in mediating leptin effects. These authors hypothesized that leptin might influence the synthesis and release of cumulus cell-derived factors, which reach the oocyte through gap junction coupling and/or the extracellular environment.

Leptin acts via transmembrane receptors, which show structural similarity to the class I cytokine receptor family. The leptin receptor (Ob-R) is produced in several alternatively spliced forms that have in common an extracellular domain of over 800 amino acids, a transmembrane domain of 34 amino acids and a variable intracellular domain, characteristic for each of the isoforms. These isoforms can be classified into three main classes: short (Ob-Ra), long (Ob-Rb) and secreted [8].

In the mouse, Ryan et al. [9], using immunohistochemistry, observed protein expression of the long form of the leptin receptor (Ob-Rb) in the ovary, with high intensities observed in oocytes, thecal cells and corpora lutea with peak expression at ovulation. In the pig, Craig et al. [5] demonstrated that Ob-R is expressed in oocytes from all stages of follicular development and oocyte maturation, with the highest level of expression occurring in oocytes from medium follicles and at GVBD, indicating that its expression is dependent on follicular stage and oocyte maturation.

In the horse, *in vitro *fertilization (IVF) has been for a long time unsuccessful and reasons have been related to incomplete *in vitro *oocyte maturation (IVM) [10], inefficient sperm capacitation [11] or changes in oocyte zona pellucida [12,13]. In a recent study, McPartlin et al. [14] characterized stallion sperm hyperactivation and demonstrated that hyperactivation of capacitated sperm supported equine IVF. Intracytoplasmic sperm injection (ICSI) has been adopted as an alternative method to conventional IVF because sperm injection eliminates problems related to sperm binding and penetration but the complexity of oocyte maturation has not yet been overcame. ICSI is a valid tool for evaluating cleavage rates of *in vitro*-matured horse oocytes and ooplasmic maturation. Several studies reported a cleavage rate of 50-80% [15-18]. Unfortunately, only a small percentage of cleaved zygotes goes on to form blastocysts in culture (25-35%) [19,20]. This result may reflect the poor cytoplasmic maturation of equine oocytes matured *in vitro *[21]. In the literature, different culture media have been evaluated to improve the rate of equine oocyte maturation, including TCM199 [22-27], B2 [22] and Ham's F10 [28], supplemented with different concentrations of serum, hormones or follicular fluid. These conditions resulted in maturation rates varying from 20 to 85% but none of these has increased the efficiency of IVF or ICSI.

The presence of leptin and leptin receptor in equine oocytes have been previously evidenced by an immunocytochemical study in compact cumulus oocytes recovered immediately upon collection and after in vitro maturation (IVM) from fillies and from mares of light or heavy body weight breeds [29]. To our knowledge, studies on the effects of leptin in equine oocytes and embryos were not reported to date. Since oocyte developmental competence is best assessed by its ability to undergo embryonic development [30], the present study investigated the effect of leptin supplementation in IVM medium on maturation, fertilization and development of horse oocytes after ICSI. In addition, the developmental expression of Ob and Ob-R proteins in early embryo development was analyzed by immunocytochemical staining.

## Methods

All chemicals were purchased from Sigma-Aldrich (Milano, Italy) unless otherwise indicated.

### Collection of oocytes

The study was conducted in Southern Italy (41° North parallel). Ovaries from mares of unknown reproductive history obtained at two local abattoirs, located at a maximum distance of 20 Km (30 min) from the laboratory, were transported and processed for the scraping procedure as previously described [31]. Cumulus-oocyte complexes (COCs) were recovered from medium size follicles (0.5 to 2.5 cm in diameter), identified in the collected mural granulosa cells by using a dissection microscope and only healthy COCs, classified as having an intact, compact (Cp) or expanded (Exp) cumulus investment [24,31] were selected for culture; degenerating oocytes showing shrunken, dense or fragmented cytoplasm were recorded and discarded. The time between follicle scraping and beginning of oocyte culture was less than 1 hour. Total time between slaughter and culture ranged between 2 to 4 hours.

### In vitro maturation

In vitro maturation (IVM) was performed following the procedure described by Dell'Aquila et al., 2003 [31]. Medium TCM-199 with Earle's salts, buffered with 4.43 mM HEPES and 33.9 mM sodium bicarbonate and supplemented with 0.1 g/L L-glutamine, 2 mM sodium pyruvate, 2.92 mM calcium-L-lactate penthahydrate (Fluka 21175 Serva Feinbiochem GmbH & Co Heidelberg, Germany No.29760) and 50 μg/mL gentamicin was used. After preparation, pH was adjusted to 7.18 and the medium was filtered through 0.22-μm filters (No.5003-6, Lida Manufacturing Corp., Kenosha WI, USA) and stored/refrigerated (4°C) until use for a maximum of one week. On the day of IVM, medium was further supplemented with 20% (v/v) Fetal Calf Serum (FCS). Then, gonadotrophins (10 μg/mL ovine FSH and 20 μg/mL ovine LH) and 1 μg/mL 17β Estradiol were added. The medium was filtered again and allowed to equilibrate for 1 hour under 5% CO_2 _in air before being used. Compact and expanded COCs were washed three times in the culture medium and groups of up to 10 COCs with the same cumulus morphology were placed in 400 μL of medium/well of a four-well dish (Nunc Intermed, Roskilde, Denmark), covered with pre-equilibrated lightweight paraffin oil and cultured for 28 to 30 h at 38.5°C under 5% CO_2 _in air. The effects of recombinant human leptin (Sigma L-4146), added to the culture well, were tested at the concentrations of 1, 10, 100 and 1000 ng/ml that were reported to be effective in stimulating oocyte maturation in dose-response curve experiments in porcine [5,6] and bovine [7] oocytes. Oocytes cultured in the absence of leptin were used as controls.

### Oocyte preparation for ICSI

After IVM culture, oocytes underwent cumulus and corona cells removal by incubation in TCM 199 with 20% FCS containing 80 IU hyaluronidase/mL and aspiration in and out of glass pipettes finely heat pulled to the diameter of the equine oocytes. Oocyte morphology after denuding was assessed under a Nikon SMZ 1500 stereomicroscope (× 60-110 magnification). Those oocytes showing an intact zona pellucida, regular-shaped perivitelline space, 1^st ^polar body presence in the perivitelline space, intact oolemma, regular ooplasmic shape and texture (no vacuoles) were classified as mature and morphologically normal [32-34] and underwent microinjection.

### Semen preparation for ICSI

Fresh semen samples from a mature stallion with a reproductive history of normal fertility were used and trials were performed in the reproductive season (February to September 2008). The stallion was located in the reproductive centre Pegasus (Department of Animal Production, University of Bari, Southern Italy) and was routinely used in artificial insemination programs. Semen was collected by using Missouri artificial vagina with an in-line gel filter, extended with INRA 96 (IMV Technologies, Piacenza, Italy) at the concentration of 20-25 × 10^6 ^sperm cells/mL and used immediately. Sperm cells for ICSI were prepared by the swim-up procedure in Earle's balanced salt solution (EBSS) supplemented with 0.4% BSA, 50 μg/mL gentamicin as previously described [31-35].

### ICSI procedure

Intracytoplasmic sperm injection was carried out as previously reported [31-35]. All procedures were performed at 38.5°C in Global medium (IVFonline, Ontario, Canada). Each injected oocyte was then transferred to a single 25 μL drop of fresh Global medium covered by lightweight paraffin oil and incubated at 38.5°C for 18-20 hours under 5% CO_2 _in air.

### Embryo culture and evaluation

Injected oocytes were allowed to further develop in vitro for 72 hours in the same medium. On each culture day, embryonic developmental stage was recorded and embryo quality was graded as follows: type a = blastomeres of equal size with <10% cytoplasm fragmentation; b = blastomeres of equal size with 10 to 40% fragmentation; c = unequal blastomeres with 10 to 40% fragmentation; d = unequal blastomeres with >40% fragmentation. At the end of culture time, embryos were removed from culture, fixed and evaluated as described below. The uncleaved ova were removed after 48 hours culture, fixed and evaluated with the same procedures as described below.

### Immunocytochemistry

According to the procedures described by Kim et al. [36], with some modifications, 2-, 4-, 8-cell stage ICSI-derived embryos, fertilized and unfertilized oocytes were fixed for 4 hours in 3.7% paraformaldehyde at 4°C. Unless otherwise stated, incubations were carried out at 4°C. Oocytes and embryos were washed four times, for 20 min, in PBS containing 1% Triton X-100 (PBS-T). *First step: *oocytes and embryos were placed overnight in a blocking solution consisting of 0.1 M glycine, 1% goat serum, 0.01% Triton X-100, 1% powdered nonfat dry milk, 0.5% bovine serum albumine (BSA) and 0.02% sodium azide in PBS. The Ob-R primary antibody was raised against a recombinant protein corresponding to amino acids 541-840 mapping within an internal domain of human Ob-R (sc-1834, Santa Cruz Biotech Heidelberg Germany). After blocking, oocytes and embryos were incubated overnight with the primary antibody diluted to 1:100 in PBS-T. *Second step: *oocytes and embryos were then washed four times, for a total time of 15 min, in PBS-T and placed in the solution containing the secondary antibody [goat anti-rabbit fluorescein isothiocyanate (FITC)-conjugated antibody, Santa Cruz Biotech, diluted 1:100 in PBS-T] for 4 h. Oocytes and embryos were washed four times, for 15 min, in PBS-T. The Ob primary rabbit antibody was raised against the N-terminal region of the Ob gene product of human and to a lesser extent, mouse and rat (sc-842; Santa Cruz Biotech. Inc., Santa Cruz, CA). Oocytes and embryos were incubated overnight with the primary antibody diluted to 1:100 in PBS-T. *Third step: *After washing four times, for a total time of 15 min, in PBS-T, oocytes and embryos were incubated with avidine for 20 min and then, after further washing, with the secondary antibody (Biotinylated Anti-Rabbit IgG made in goat: D.B.A. Vector, UK; BA-1000) for 4 hours at 4°C and with Rhodamine Avidin D, TMRITC (D.B.A. Vector, UK; A-2002) for 3 hours. Rhodamine provided a second label to FITC. For each experimental trial, two-three embryos and uncleaved oocytes were used as minus primary controls. After these steps oocytes and embryos were stained with 2.5 μg/mL Hoechst 33258 (Sigma 1155) in 3:1 (vol/vol) glycerol/PBS, mounted on microscope slides, covered with cover slips, sealed with nail polish and kept at 4°C in the dark until observation. In order to avoid excess pressure being exerted on the oocytes/embryos, the coverslides were supported with thick droplets of a Vaseline-wax mixture placed in each corner.

To test the specificity of the immunoreactions, histological sections of equine subcutaneous fat were used as positive controls.

### Nuclear chromatin evaluation

Oocytes and embryos were evaluated in relation to their developmental stage under an epifluorescence microscope (Nikon Eclipse 600 equipped with B-2 A (460 nm excitation/346 nm emission) filter as previously described [35-37]. Normally cleaved embryos were defined by the presence of nuclei of regular morphology for each blastomere. In the group of uncleaved ova, normal fertilization was defined by the presence of two polar bodies (PBs) with two pronuclei (PN). Presence of the metaphase II (MII) with the 1^st ^PB with the swollen sperm head, a single PN with signs of the sperm cell in the cytoplasm, tripronucleate zygotes with a single PB extruded, were considered to represent retarded, arrested or abnormal fertilization, respectively, and were classified and grouped as abnormally fertilized oocytes. Oocytes with one PN with intact sperm cell were regarded as activated oocytes. Oocytes showing MII+PB with an intact sperm cell were classified as unfertilized. Fertilization rates in these trials included the oocytes that developed further into embryos as well as those that were found uncleaved but with evident signs of fertilization after staining.

### Evaluation of leptin and leptin receptor expression by confocal microscopy

Oocytes and embryos were observed at 600× magnification in oil immersion with a laser scanning confocal microscope (C1/TE2000-U Nikon). An Argon laser ray at 488 nm and the B-2 A filter (495 nm exposure and 519 nm emission) was used to point out the FITC-conjugated secondary antibody for Ob-R labelling. A Helium/Neon laser ray at 543 nm and the G-2 A filter (555 nm exposure and 580 nm emission) was used to point out the TMRITC-conjugated secondary antibody for Ob labelling. Scanning was conducted with 25 optical series from the top to the bottom of the oocyte with a step size of 0.45 μm to allow three-dimensional distribution analysis. Parameters related to fluorescence intensity were maintained at constant values for all measurements.

### Statistical analysis

The statistical significance of the results was evaluated by the Chi-square-test with the Yates correction for continuity and by Fisher's exact test. Fisher's exact test was used when a value of less than 5 was expected in any cell. Proportions of matured, fertilized oocytes and cleaved embryos after ICSI were compared between each leptin-treatment group and controls. Values with *P *< 0.05 were considered to be statistically significantly different.

## Results

### Effect of leptin supplementation in IVM medium on maturation and fertilization after ICSI

Five consecutive IVM/ICSI trials were performed in the reproductive season with the aim to evaluate the effects of leptin supplementation in IVM medium on maturation, fertilization and developmental potential of equine oocytes. The ovaries of 60 mares were processed, 503 follicles were scraped and 283 oocytes were recovered (2.4 oocytes/ovary; 57%, n° of recovered oocytes/n° scraped follicles), 149 surrounded by a Cp cumulus and 134 with an Exp cumulus. After culture and cumulus removal, 262 oocytes (93%), 137 Cp and 125 Exp, were found as morphologically normal and analyzed for maturation (1^st ^PB extrusion). Of them, 62 Cp and 77 Exp oocytes were found matured (total = 139 oocytes), were submitted to ICSI and allowed to develop in vitro for 72 hours after sperm injection.

Table 1 shows the maturation and fertilization rates, after ICSI, of oocytes cultured in presence of leptin in IVM medium. In Exp oocytes, the maturation rate was significantly higher in 100 ng/ml leptin-treated oocytes compared with controls (17/23, 74% vs 17/39, 44%; P < 0.05). In the group of Cp oocytes, the proportion of matured oocytes did not differ between leptin-treated and control oocytes. In both groups, Cp or Exp oocytes, there were no statistically significant differences between groups with respect to the percentages of normally, abnormally fertilized or activated oocytes. However, the total (normal + abnormal) fertilization rate was significantly higher in 10 ng/ml leptin-treated Exp oocytes compared with controls (9/16, 56% vs 9/39, 23%; percentages of evaluated oocytes, P < 0.05).

**Table 1:** Dose response effect of leptin on in vitro maturation and fertilization rates of equine oocytes after ICSI

				**N° (%) of oocytes**			
**Cumulus morphology**	**Leptin concentration (ng/ml)**	**Evaluated**	MII+PB and injected*	Normally fertilized*	Abnormally fertilized*	Total fertilization*	Activated*
Compact	0	40	17 (43)	11 (28)	0 (0)	11 (28)	0 (0)
	1	20	11 (55)	6 (30)	0 (0)	6 (30)	1 (5)
	10	26	13 (50)	8 (31)	0 (0)	8 (31)	1 (4)
	100	25	9 (36)	5 (20)	1 (4)	6 (24)	1 (4)
	1000	26	12 (46)	5 (19)	1 (4)	6 (23)	1 (4)
Expanded	0	39	17 (44)^a^	9 (23)	0 (0)	9 (23)^a^	2 (5)
	1	23	14 (61)	8 (35)	0 (0)	8 (35)	0 (0)
	10	16	12 (75)	8 (50)	1 (6)	9 (56)^b^	0 (0)
	100	23	17 (74)^b^	7 (30)	2 (9)	9 (39)	1 (4)
	1000	24	17 (71)	9 (38)	1 (4)	10 (42)	0 (0)
Total	0	79	34 (43)	20 (25)	0 (0)	20 (25)	2 (3)
	1	43	25 (58)	14 (33)	0 (0)	14 (33)	1 (2)
	10	42	25 (60)	16 (38)	1 (2)	17 (40)	1 (2)
	100	48	26 (54)	12 (25)	3 (6)	15 (31)	2 (4)
	1000	50	29 (58)	14 (28)	2 (4)	16 (32)	1 (2)

(*) percentages of the evaluated oocytes

Percentages with different superscripts statistically differ at *P *< 0.05 (Chi-square-test).

### Effects of leptin supplementation in IVM medium on in vitro embryo development

Table 2 shows the cleavage rates after ICSI of oocytes cultured in presence of leptin in IVM medium. The addition of leptin during IVM culture was not effective on embryonic development at the 2-4 cell stage. The rates of embryos which cleaved at the 2-4 cell stage did not statistically differ between leptin treated and control samples (percentages of the 2-cell stage embryos, NS). However leptin, added at the concentrations of 100 ng/ml, significantly reduced the rates of embryos reaching the 4-8 cell stage (1/11, 9% vs 10/19, 53%; percentages of the 2-cell stage embryos, P < 0.05). Whether calculated in respect to the number of evaluated oocytes, the effects of leptin did not attain statistical significance.

**Table 2:** Effects of leptin added to IVM medium on equine embryo development

			**N° (%) of oocytes**			
**Cumulus morphology**	**Leptin concentration (ng/ml)**	**Evaluated oocytes**	N° (%) of 2 cell stage embryos**	N° (%) 4 cell stage embryos*	N° (%) 8 cell stage embryos*	N° (%) 8 cell stage embryos**
Compact	0	40	11 (28)	10 (91)	5 (45)	5 (13)
	1	20	6 (30)	6 (100)	5 (83)	5 (25)
	10	26	7 (27)	6 (86)	2 (29)	2 (8)
	100	25	5 (20)	5 (100)	0 (0)	0 (0)
	1000	26	5 (19)	4 (80)	0 (0)	0 (0)
Expanded	0	39	8 (21)	7 (88)	5 (63)	5 (13)
	1	23	8 (35)	8 (100)	5 (63)	5 (22)
	10	16	6 (38)	5 (83)	4 (67)	4 (25)
	100	23	6 (26)	4 (67)	1 (17)	1 (4)
	1000	24	9 (38)	7 (78)	2 (22)	2 (8)
Total	0	79	19 (24)	17 (89)	10 (53)^a^	10 (13)
	1	43	14 (33)	14 (100)	10 (71)	10 (23)
	10	42	13 (31)	11 (85)	6 (46)	6 (14)
	100	48	11 (23)	9 (82)	1 (9)^b^	1 (2)
	1000	50	14 (28)	11 (79)	2 (14)	2 (4)

(*) percentages of the two cell-stage embryos

(**) percentages of the evaluated oocytes

Different superscripts indicate statistically differences at *P *< 0.05 (Chi-square-test).

Embryo quality did not differ between controls and 1, 10 and 1000 ng/ml-treated oocytes. Instead, the exposure to 100 ng/ml significantly increased the rate of embryos, issuing from Exp oocytes, with grade b of cytoplasmic fragmentation. In detail, in control oocytes, 9 (82%) out of the 11 embryos from Cp oocytes and 6 (75%) out of the 8 embryos from Exp oocytes were categorized as type a; in the group of oocytes treated with 1 ng/ml, 5 (83%) out of the 6 Cp embryos and 6 (75%) out of the 8 Exp embryos were categorized as type a; in oocytes treated with 10 ng/ml, 4 (57%) out of the 7 Cp embryos and 4 (66%) out of the 6 Exp embryos were categorized as type a; in oocytes treated with 100 ng/ml, 3 (60%) out of the 5 Cp embryos but none of the 6 Exp embryos were categorized as type a; in oocytes treated with 1000 ng/ml, 3 (60%) out of the 5 Cp embryos and 7 (78%) out of the 9 Exp embryos were categorized as grade a. In all experimental groups, the remaining embryos were of grade c except a Cp embryo from 100 ng/ml which resulted of grade d.

### Immunolocalization of Ob and Ob-R in equine early embryos

**Figure 1:**
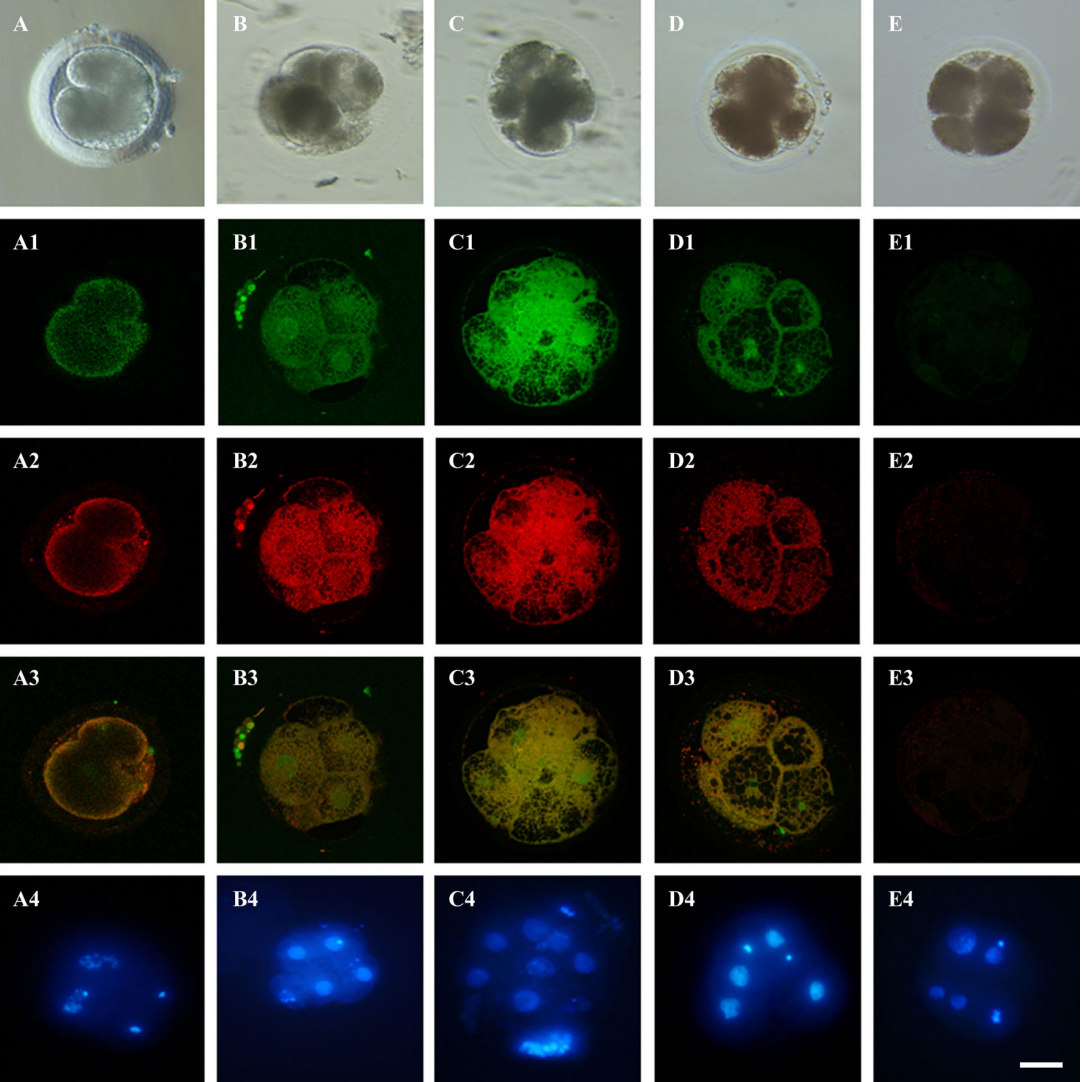
**Immunocytochemical analysis of Ob and Ob-R proteins expression**. Representative confocal images of embryos obtained by ICSI in oocytes matured in vitro, stained with antibodies directed against Ob-R and Ob protein. For each raw, corresponding bright-field (A, B, C, D, E), confocal (A1, B1, C1, D1, E1 for Ob-R; A2, B2, C2, D2, E2 for Ob labeling and A3, B3, C3, D3, E3 for Ob-R and Ob merge) and UV light images of blastomere nuclei (A4, B4, C4, D4, E4) of the same embryo are shown. After Hoechst 33258 staining, to evaluate chromatin configuration, nuclei of regular morphology in each blastomere and residual polar bodies are visible. The 2-cell stage embryo in raw A is representative of embryos issuing from oocytes cultured in presence of 1000 ng/ml leptin; the 4-cell stage embryo in raw B was cultured in presence of 1 ng/ml leptin and the 8-cell stage embryo in raw C was cultured in presence of 10 ng/ml leptin; the 4-cell stage embryo in raw D was cultured in absence of leptin; the 4-cell stage embryo in raw E represents the negative control (no primary antibodies against Ob and Ob-R). Scale bar represent 60 μm.

**Figure 2:**
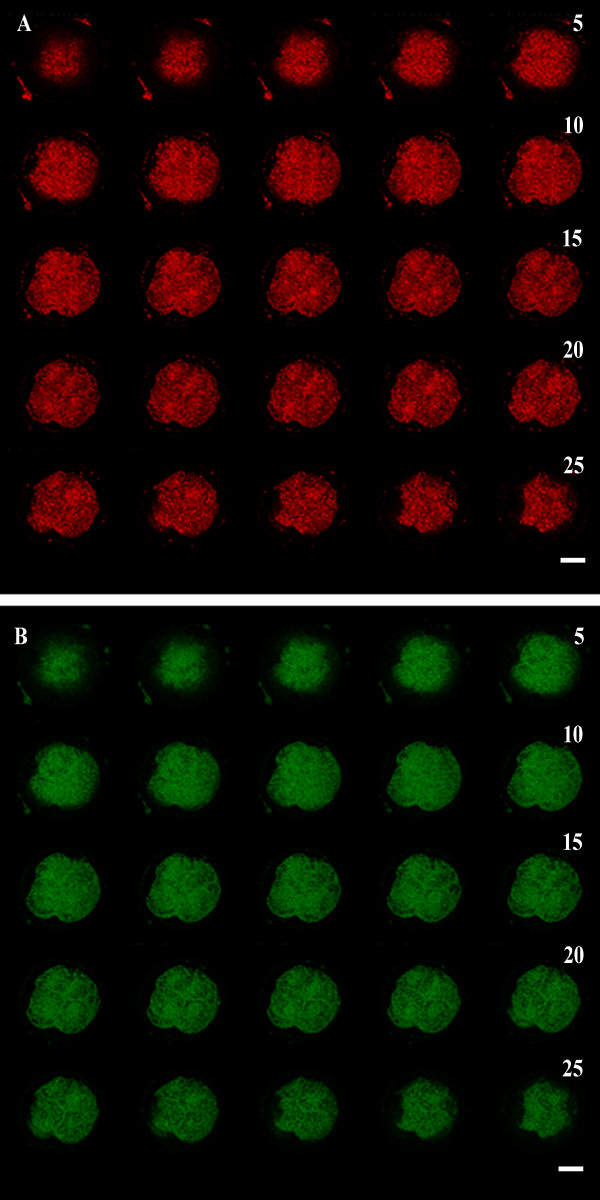
**Subcellular localization of Ob-R (A) and Ob (B) proteins in an equine IVM/ICSI-derived embryo**. The sample is representative of confocal investigations on 25 serial optical sections (step size = 0.45 μm) performed on all examined embryos. A 4-cell stage embryo is shown obtained after IVM culture in presence of 100 ng/ml leptin. In surface planes, in the lower (planes n° 1 to 5) and upper (planes n° 19 to 25) parts of the embryo, a labeling in clumps/clusters can be seen while in planes nearer the equatorial position (planes n° 6 to 18), an intense fluorescence is observed beneath the oolemma together with uniform distribution of Ob (A) and Ob-R (B) staining within the cytoplasm. Scale bar represent 60μm.

Both leptin ligand and receptor proteins were detected in embryos obtained from Cp and Exp oocytes. Both proteins were detected at the 2- (Figure 1A1, A2), 4- (Figure 1B1, B2) and 8-cell stage (Figure 1C1, C2) and were overlapped and localized in the same area (Figure 1A3, B3, C3). Figure 2 shows a representative 25 optical planes analysis of an embryo obtained after IVM culture in presence of 100 ng/ml leptin. At all analyzed stages, Ob and Ob-R were present with cortical distribution in each blastomere over the 25 optical planes. Moreover, a granule-like expression pattern was observed in the cytoplasm of each blastomere. Leptin receptor staining was positive in the nuclei of the 4- and 8-cell embryos (Figure 1B1, C1, D1). The addition of leptin in culture medium did not modified Ob and Ob-R proteins subcellular localization in equine early embryos. The same cortical pattern was evident in mature uncleaved fertilized and unfertilized oocytes. No immunoreactivities were detected in the negative controls embryos where primary antibodies were omitted. Moreover, the reactions of the tissues used as positive controls gave the expected results (equine subcutaneous adipose tissue; data not shown).

## Discussion

Our results demonstrated that the addition of leptin in the range between 10 and 1000 ng/ml increased the maturation rate of equine oocytes even though the statistical significance was observed only at the concentration of 100 ng/ml. This result is in line with previous observation in other species [5-7]. The improvement of maturation rate of oocytes may be related to some potential action mechanisms exerted by leptin on oocyte cytoplasmic maturation. These mechanisms may include direct or indirect cumulus cell-mediated effects such as restructuring oocyte cytoskeleton, reprogramming protein synthesis, or inhibiting apoptosis [38]. As previously observed in bovine, it can be hypothesized that leptin may rescue oocytes that could potentially undergo apoptosis [30]. The beneficial effect of leptin during oocyte maturation suggests a role for leptin as a survival factor minimizing cellular damage to oocyte and/or cumulus cells.

The different response to leptin treatment, observed between Cp and Exp oocytes, could be due to Ob-R modifications occurring during the process of cumulus expansion and/or to different expression or activation status of the receptor in COCs of these two categories. Previous studies, reporting concentration and stage dependent effects of leptin on embryonic development may support our hypothesis [39] and it is possible that Ob-R could activate in different ways the reported signal-transduction pathways [8] in Cp and Exp oocytes. Moreover, it has been suggested that leptin may induce germinal vesicle breakdown (GVBD), *in vivo*, via its action on the theca cells [9]. On the contrary, *in vitro*, the effects of leptin on oocyte maturation may be exerted by a direct action on the oocyte or indirect effect on cumulus cells. Leptin may influence the synthesis and release of cumulus cell-derived factors, which reach the oocyte through gap junction coupling and the extracellular environment, in different way in Cp or Exp oocytes and, consequently, it can be hypothesized that Exp cumulus cells could be more responsive to leptin than Cp cumulus cells [7].

Fertilization rate and embryonic developmental competence (cleavage and blastocyst rate) are widely used as indicators of oocyte quality. The enhanced fertilization rate observed in leptin-treated oocytes confirmed the stimulatory effect of leptin on oocyte quality. In contrast with data reported in other species, such as pig [5,6] and bovine [7], in the present study, leptin had no beneficial effect on cleavage rates after ICSI but rather, at the concentration of 100 ng/ml, it decreased embryonic developmental rate and increased cytoplasmic fragmentation. Landt et al. [40] reported that leptin plasma levels differ between various strains of rats, with variation up to two times, suggesting that different genetic background can affect circulating leptin levels. It can, therefore, be supposed that different thresholds may exist in different subjects, cells and tissues, including oocytes and embryos, with respect to leptin sensitivity, in different species. Few information is available about intrafollicular leptin concentration in differents species. In women with intrafollicular leptin concentrations equal to or higher than 20 ng/ml, the fertilization rate is significantly higher (85.7%) than that in women with lower doses (16.7% p < 0.05). No differences were detected, instead, in the quality of the embryos obtained either at the zygote stage or 48 hours after oocyte insemination [41,42]. In pigs, leptin was detected in follicular fluids pooled from different size follicles as follows: small follicles, 1.21 ± 0.28 ng/ml; medium follicles, 1.24 ± 0.06 ng/ml; and large follicles, 1.13 ± 0.24 ng/ml and when leptin was added in the maturation medium at the concentration of 10 ng/ml significantly increased the proportion of oocytes that reached the MII stage after 48 h IVM: this concentration should still be considered as close as possible to physiological levels [5]. To our knowledge, no data on leptin concentration in the follicular fluid is available in the horse and it could be possible that the concentration of 100 ng/ml do not respect the physiological condition. In addition, the Ob/Ob-R system could significantly differ in the horse compared with humans, non-human primates [43] and other species. Another possible explanation could be the different types of leptin used in various experiments as reported by Herrid et al. [39]. We used recombinant human leptin which, by bioinformatic comparison, exhibited high homology to the leptin of bovine (84%), pig (85%), equine (82.7%) and mouse (83%). Previously reported studies [5-7,30] also used recombinant human leptin. Moreover, caution must be taken when comparing different studies due to extensively different culture conditions dependent on the examined species. Again, our apparently contraddictory results may be due to differences in semen source (frozen-thawed versus fresh) or fertilization procedures. In our study, ICSI procedure with fresh semen in Global medium was previousy tested as a reliable method to obtain equine embryos [44] whereas in pigs, Kim et al. [36] and Kun et al. [6] used IVF and Somatic Cell Nuclear Transfer embryo (SCNT) in NCSU (North Carolina State University) medium; in bovine, IVF in SOF (Synthetic Oviductal Fluid) medium was adopted [7].

Our results demonstrated that Ob and Ob-R proteins were detected in equine ICSI embryos throughout early cleavage stages. This finding is in agreement with previous observation in other species [36]. Moreover, leptin has been found to be secreted by various reproductive organs including placenta [45,46] and ovary [47]. Leptin has been reported to be expressed at high levels in mouse oocytes at all stages of follicular development whereas low expression levels were found in the mural granulosa, stroma, theca, and corpora lutea [3]. Leptin receptor mRNA and protein were present in the mouse oocytes [48] and preimplantation embryos [3]. It has been reported that cultured human blastocysts secrete leptin, and the levels of leptin are significantly higher than those of arrested embryos [49]. In human, leptin protein was localized in immature oocytes and in all stages of embryonic development [50,51]. Recently, leptin protein was reported to be expressed in all stages of porcine IVF embryos [52]. This finding is also in agreement with our previous observation in equine oocytes. In oocytes at the GV stage [29], both Ob and Ob-R were uniformly distributed throughout the ooplasm, but the intensity of reaction was lower either in light weight mares or in fillies oocytes, than in oocytes of heavy weight mares. In matured oocytes, both Ob and Ob-R were localized in the cortex and concentrated at one pole of the oocyte. This distribution was indipendent from the animal group and again with lower intensity in light mares and fillies.

Leptin and Ob-R proteins in equine embryos were distributed according to the same cortical and cytoplasmic granule-like distribution pattern in each blastomere. Interestingly, positive staining was also observed in the nuclei of 4- and 8-cell stage embryos. This finding is in agreement with previous observations of nuclear positivity in neurons in rat brain [53] and perinuclear positivity in transfected Ob-R expressing HeLa cells [54]. This latter study [54] examined the intracellular traffic of Ob-R and reported that both isoforms of Ob-R were observed in HeLa cells at three cellular localizations, the plasma membrane, the peripheral cytoplasm and the perinuclear compartment. The perinuclear staining, localized in the trans Golgi network area, was reported as probably made of newly synthetized receptors en route to the cell surface [54]. The antibody for Ob-R used in the present study detects both short and long forms of Ob-R [55]. Thus, it is not known which Ob-R isoform mediated the effect of leptin on equine oocytes during IVM and is expressed in equine embryos.

## Conclusion

The present study demonstrated for the first time that, in the horse, the addition of leptin during IVM, in the range between 10 and 1000 ng/ml, has a beneficial effect on meiotic maturation and fertilization after ICSI but it impairs embryonic development. In addition, it was demonstrated that Ob and Ob-R proteins are expressed in equine early embryos. The presence of both ligand and receptor proteins in oocytes [29] and in ICSI embryos suggests that leptin acts as an autocrine/paracrine hormone in horse maturation, fertilization and early development. Species-specific differences may exist in oocytes/embryos with regard to the sensitivity to leptin.

## Competing interests

All authors declare that there is no financial or non-financial competing interest that could be perceived as prejudicing the impartiality of the reported research.

## Authors' contributions

ALC, MED, GML and FC conceived and designed the study. MED carried out and coordinated IVM/ICSI/embryo culture experimental procedures. ALC carried out and coordinated the early developmental expression study. NF and BA performed the in vitro cultures and evaluations of oocytes and embryos. GML performed semen collection and preparation for ICSI. YSC coordinated the ICSI and early embryo culture setting. MED and BA performed ICSI procedures. GB helped in setting up the immunocytochemical study. ALC, NF and BA performed the immunocytochemical procedures. BA performed the evaluations by confocal microscopy and figures preparation. SA participated in experiment supervision and in critical reading of the manuscript. NF and BA performed nuclear stage evaluation, Ob/Ob-R receptor expression evaluation and the statistical analysis. ALC and MED wrote the manuscript. All the Authors read and approved the final manuscript.
